# CircRNA ZNF609 promotes the growth and metastasis of thyroid cancer *in vivo* and *in vitro* by downregulating miR-514a-5p

**DOI:** 10.1080/21655979.2022.2033015

**Published:** 2022-02-08

**Authors:** Ping Shi, Yan Liu, Dongqiang Yang, Yanzhao Wu, Lan Zhang, Shanghua Jing, Huijing Shi, Cuizhi Geng

**Affiliations:** a Department of Otolaryngology Head and Neck Surgery, Hebei Medical University Fourth Affiliated Hospital and Hebei Provincial Tumor Hospital, Shijiazhuang, China; b Department of Radiological Intervention, Hebei Medical University Fourth Affiliated Hospital and Hebei Provincial Tumor Hospital, Shijiazhuang, China; c Department of General Surgery, Hebei Medical University Fourth Affiliated Hospital and Hebei Provincial Tumor Hospital, Shijiazhuang, China

**Keywords:** Thyroid cancer, CircRNA ZNF609, miR-514a-5p, proliferation, migration and invasion

## Abstract

Thyroid cancer (TC) often manifests in the form of a painless cervical mass or nodule and continues to increase in incidence. Currently, much less is known about its pathogenesis in TC cells. This study sought to figure out what role the circular RNA (circRNA) ZNF609/miR-514a-5p might play in TC development and metastasis. In this study, the detection of circ-ZNF609 and miR-514a-5p expressions was carried out by qRT-PCR in TC cell lines. Cell proliferation assessment is employed by cell counting kit-8 (CCK-8) assay, colony formation, Western blot and immunofluorescence. Cell invasion and migration measurement were conducted applying wound healing and transwell. The relationship between circ-ZNF609 and miR-514a-5p was subjected to prediction with bioinformatics analysis and validated with the aid of luciferase reporter assay. Furthermore, xenograft animal experiment was adopted to confirm the role of circ-ZNF609/miR-514a-5p in TC *in vivo*. The data indicated that circ-ZNF609 was highly expressed, while miR-514a-5p was downregulated in TC cells. Circ-ZNF609 knockdown prevented the malignant biological behaviors of TPC-1 and IHH-4 cells. Besides, circ-ZNF609 sponged miR-514a-5p and miR-514a-5p knockdown reversed the suppressed impacts of circ-ZNF609 knockdown on TC cell malignant biological behaviors. In addition, the silence of circ-ZNF6091 significantly repressed, whereas miR-514a-5p silencing promoted TC tumorigenesis *in vivo*. The findings highlighted the importance of circ-ZNF609 function in facilitating TC cell development and metastasis *in vitro* and *in vivo* via binding to miR-514a-5p.

## Graphical abstract

**Figure:**
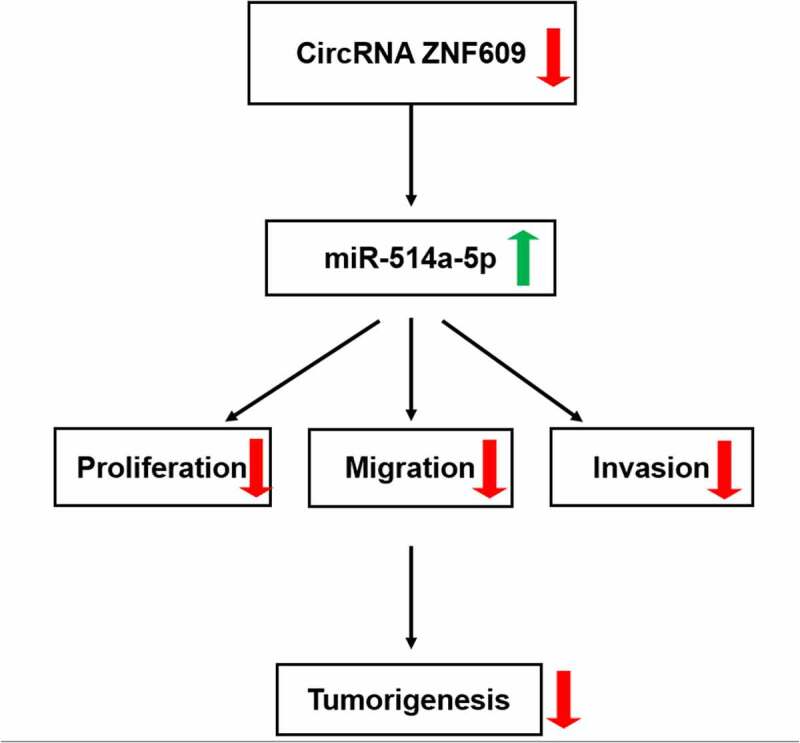

## Introduction

Thyroid cancer (TC) stands out as one of the world’s predominant malignancy in endocrine system [1,2]. In recent years, the morbidity of thyroid cancer has been growing much faster than other cancers [3]. TC is a kind of indolent cancer exhibiting no obvious symptoms at an early stage and some specific symptoms at an advanced stage, for example, hoarseness, difficulty swallowing and Horner syndrome caused by sympathetic nerve compression [4]. Despite the advances in treatments including targeted biologic therapy and adjuvant radiotherapy, the total rate of TC patient surviving successfully is not ideal for the reason of local recurrence and distant metastasis [5]. Thus, discovering effective therapeutic target and identifying potential mechanism in tumorigenesis of TC are significantly imperative.

Noncoding RNAs (ncRNAs) are divided into linear ncRNAs and circular RNAs (circRNAs) based on different structures [6]. In the last few decades, linear ncRNAs including microRNAs (miRNAs) and lncRNAs have reached more attention because circRNA is relatively less and was once regarded as a by-product of mis-splicing [7]. It was not until 2015 that circRNA was reported to be widely existed in animals, participating in a variety of biological process and disease pathogenesis [8,9]. To name a few, Han et al. revealed that circ-MTO1 was lowly expressed in and inhibited malignant progression of hepatocellular carcinoma [10]. Sun et al. demonstrated that circ-SFMBT2 was upregulated in gastric cancer tissues and exacerbated the proliferation of gastric cancer cells by acting as a sponge of miR-182-5p to enhance CREB1 expression [11]. Additionally, dysfunctional circRNAs like circ-ITCH, circRAPGEF5, circ_0025033, circZFR and circRNA_102171, was also found in thyroid cancer, indicating the critical contribution of circRNAs in thyroid cancer pathogenesis [12]. Circ-ZNF609 (ID: hsa_circ_0000615 in circBase), located at chr15: 64,791,491–64,792,365, has been reported to exert important biological roles in promoting the tumorigenesis and metastasis of multiple types of cancers [13]. Du et al. reported that circ-ZNF609 promoted glioma growth and metastasis via enforcing PLK1 expression by competitive binding to miR-1224-3p and aggravate malignant progression of glioma [14]. However, the role of circ-ZNF609 in the occurrence and metastasis of thyroid cancer is still needed for further exploration. Previous studies have shown that the downregulation of miR-514a-5p expression participates in tumor malignant properties [15]. Li et al. noted that circular RNA circ-CCAC1 induced adrenocortical carcinoma cell malignant biological behaviors via sponging miR-514a-5p and targeting C22orf46 [16]. In addition, the long noncoding RNA TRIM52-AS1 sponges miR-514a-5p to promote the progression of hepatocellular carcinoma by increasing MRPS18A [17].

In this study, we aimed to identify the role and mechanism of circ-ZNF609 in TC, and we hypothesized that circ-ZNF609 may bind to miR-514a-5p to inhibit cell proliferation, metastasis, and tumor growth, which may pose a new prospective target for the therapy of TP.

## Materials and methods

### Cell culture

Normal thyroid cell-line Nthy-ori 3–1 and the TC cell lines CGTH-W3, TPC-1, IHH-4 CAL-62 and B-CPAP (Chemicalbook, Beijing, China) were grown in DMEM (Promega, Madison, WI, U.S.A.) supplemented with 10% FBS and 1% penicillin/streptomycin. The incubator temperature was set to 37°C and provided with 5% CO_2_.

### Cell transfection

The specific shRNA targeting circ-ZNF609 (sh-circ-ZNF609-1/2) and corresponding control shRNA (sh-NC) were constructed by Fitgene (Guangzhou, China). miR-514a-5p inhibitor plasmids (miR-514a-5p inhibitor) and negative control plasmid (inhibitor-NC) were constructed by ABlife (Wuhan, China). The vectors were subjected to transfection into TPC-1 or IHH-4 cells with the aid of Lipofectamine 2000 (Invitrogen, CA, USA) as per the recommendations instructed by vendor [18].

### Quantitative real-time polymerase chain reaction (qRT-PCR)

The extraction of total RNA was undertaken with the application of TRIzol reagent (Invitrogen, Carlsbad, CA, USA). A NanoDrop 3000 spectrophotometer (ThermoScientific, Waltham, MA) was used to confirm the quality and concentration of total RNA following the standard procedures recommended by the supplier. Reverse transcription (RT) of first-strand cDNAs was conducted employing Hifair® II 1st Strand cDNA Synthesis Kit (Yeasen, Shanghai, China). Amplification of the cDNA was performed by real-time qPCR using the SYBR Premix Ex Taq™ II (Tli RNaseH Plus) kit bought from Wuhan Khayal Bio-Technology (Wuhan, China). The primer sequences for PCR are presented as follows: circ-ZNF609: 5′-CAGCGCTCAATCCTTTGGGA-3′ (forward) and 5′-GACCTGCCACATTGGTCAGTA-3′ (reverse); miR-514a-5p: 5′-TCACTACTCTGGAGAGTGACAA-3′ (forward) and 5′-GTGCAGGGTCCGAGGT-3′ (reverse); GAPDH: 5′-GGGAAACTGTGGCGTGAT-3′ (forward) and 5′-GAGTGGGTGTCGCTGTTGA-3′ (reverse). The 2^−ΔΔCT^ method was applied for the determination of the relative quantification, and the relative mRNA level was normalized with GAPDH or U6 level [19].

### Cell Counting Kit-8 (CCK-8) assay

The TPC-1 and IHH-4 cells subjected to transfection were placed in 96-well plates at a density of 5 × 10^3^ cells/well and grown in DMEM with 10% FBS by incubation together for 24 h, 48 h and 72 h at 37°C. At every time point, 10 μL of WST-8 (Beyotime, Haimen, China) was added to each well and allowed to incubate for 2 hours. Finally, the absorbance reading of each well was detected with microplate reader at 450 nm (Reagen, Shenzhen, China).

### Colony formation assay

The post-transfection cells were loaded (500 cells/well) in 6-well plates by incubating in DMEM with 10% bovine calf serum at 37°C for 2 weeks. Two weeks later, the cells were fixed with 4% paraformaldehyde and stained with 0.1% crystal violet. At last, colonies were imaged and counted by a light microscope (Precise, Beijing, China). The number of colonies, defined as >50 cells/colony, was counted.

### Wound healing assay

Cells were kept in 6-well plates for growth until achieving 80–90% confluence. A 20-μl pipette tip was used to make a straight scratch, and the medium was replaced with a serum-free DMEM medium. After 24 h incubation, cell migration into the wound surface and the number of migrated cells were counted under an inverted microscopy. Five areas in each group were selected for counting randomly [20].

### Cell invasion assay

Cell invasion assessment employed a transwell experiment [21]. In brief, transfected TPC-1 and IHH-4 cells were resuspended in a serum-free medium and loaded into the upper compartment with 0.1 mL of BD Matrigel ^TM^ supplied by Corning (NY, USA). Meanwhile, a medium with 10% FBS was added to the lower layer of the chamber. 24 h after incubation, the non-invaded cells in the upper part of the transwell membrane were removed with a sterile cotton swab. Cell underwent fixation with formaldehyde and staining with hematoxylin and eosin on the lower face and then counted under a microscope.

### Immunofluorescence staining

Immunofluorescence staining was carried out to detect the expression of Ki-67 in TPC-1 and IHH-4 cells [22]. Simply speaking, the fixation (4% polyoxymethylene) and permeabilization (0.5% Trition X-100) of two cell lines were performed before overnight incubation with primary antibody Ki-67 (1:200, ab243878, Abcam) at 4°C. Then, 1 h incubation of cells with secondary antibody (1:400, ab150077, Abcam) was undertaken at room temperature. Subsequent cell observation was carried out with an LSM 800 confocal laser scanning microscopy (Zeiss, Beijing, China).

### Western blot analysis

The total proteins were extracted from transfected cells by the use of RIPA Lysis Buffer (Elabscience, Wuhan, China) on ice. Then, the separation of protein samples employed 10% SDS-PAGE (Bio-Rad, Hercules, CA) and protein transfer to PVDF (Millipore, USA) were processed. Being Blocked with 5% skimmed milk, the overnight incubation with primary antibodies targeting Ki-67 (1:1000, ab16667), PCAN (1:1000, ab92552), MMP-2 (1:1000, ab92536), MMP-9 (1:1000, ab76003), GAPDH (1:1000, ab8245) was conducted sequentially overnight at 4°C. After three times of washes, HRP conjugated secondary antibody (Cell Signaling Technology) was utilized to cultivate the membranes for 1 h. Finally, the observation of immunoreactive protein bands employed an ECL detection system (Amersham Pharmacia Biotech, Piscataway, NJ, USA) and subjected to analysis with the aid of ImageJ software (NIH, Bethesda, MD, USA).

### Luciferase reporter assay

To create a luciferase plasmid containing the wild-type sequence of circ-ZNF609 3′UTR, the DNA sequence of circ-ZNF609 3′-UTR that included the putative miR-514a-5p binding sites was subcloned into a pGL3 vector (Promega, Madison, WI). Subsequently, the mutant circ-ZNF609 3′UTR sequence including mutated miR-514a-5p binding sites was subcloned into the pGL3 vector to create the mutant reporter vector. Afterward, the co-transfection of TPC-1 and IHH-4 cells with luciferase reporter vectors was conducted by means of lipofectamine 2000 (Invitrogen). The normalization of Luciferase activities to Renilla luciferase was carried out [23].

### Xenograft experiments

All experiments were undertaken in strict compliance with the rules established by the Animal Care and Use Committee of Hebei Medical University Fourth Affiliated Hospital and Hebei Provincial Tumor Hospital and were approved by this committee. Thirty four-month-old female BALB/c nude mice were supplied by the Laboratory Animal Center of China. The mice were kept in a 22 ± 1°C environment that provided a 12:12-hour light–dark cycle and sterilized food and water that was freely consumed. Mice were injected subcutaneously with cell suspension (1 × 10^6^ cells/100 μl) on flanks under an anesthetic with 2% pentobarbital (50 mg/kg). The size of the tumor was monitored weekly and were examined with a caliper and calculated with the use of the formula: volume = (length × width^2^)/2. Six weeks later, the mice suffocated in CO_2_ and subsequently died from cervical dislocation. The tumor tissues were then weighed and used for immunohistochemistry and Western blot detection [24].

### Immunohistochemistry

Cancer tissues from mice underwent a series of steps including fixation in 4% paraformaldehyde, dehydration, embedding in paraffin, and finally cutting into 5 µm slices. These slices were microwaved in citrate-buffered solution (pH 6.0) over 3 min for antigen retrieval. Following a 30 min block with 10% goat serum at room temperature, the sections and Ki-67 antibody (1:300, Ab15580) were co-incubated overnight at 4°C before the incubation with secondary antibody (HRP-labeled goat anti-rabbit, 1:1000, Abcam) for 30 min. The slices were exposed to DAB kit and stained with hematoxylin. The viewing of the images was carried out with the application of a light microscope (Leica Microsystems Inc., Illinois, USA).

### Statistical analysis

The analysis of all experimental data in this paper was done by SPSS 23.0 (IBM, Armonk, NY, USA). The statistical significance of the two-group comparisons was assessed by a Student’s t-test, multiple comparisons by one-way ANOVA and Bonferroni post hoc test. Data were recorded in the manner of means ± SD. A P-value below 0.05 was selected showing that the results were significant.

## Results

In this study, we investigated the biological function of circ-ZNF609 and the potential mechanism in TC cells and xenograft mice with TC. The data showed that the silencing of circ-ZNF609 suppressed TP cell proliferation, migration and invasion and restrained tumor growth in xenograft mouse model. In addition, circ-ZNF609 was found to be combined with miR-514a-5p. Moreover, miR-514a-5p silencing reversed the effects of circ-ZNF609 silencing on TC cell growth and metastasis, as well as tumor growth.

### Silencing of circ-ZNF609 inhibits the proliferation of TC cells

**Figure 1.**
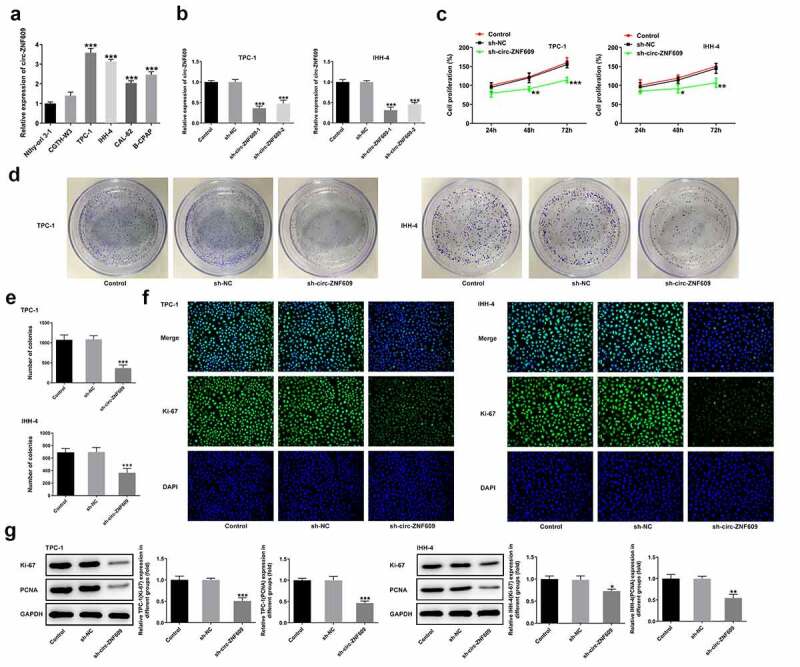
Inhibition of circ-ZNF609 inhibited the proliferation of thyroid cancer cells. (a) Relative expression of circ-ZNF609 in several TC cell lines and normal thyroid cell line Nthy-ori 3–1, examined with the application of qRT-PCR. (b) Relative expression of circ-ZNF609 in TPC-1 and IHH-4 cells following circ-ZNF609 knockdown. Cell proliferation of TPC-1 and IHH-4 transfected with sh-circ-ZNF609, assayed with the use of CCK-8 assay (c) and colony formation assay (d-e). (f) Ki-67 expression in TPC-1 and IHH-4 transfected with sh-circ-ZNF609, examined applying immunofluorescence staining. Original magnification: 200 × . (g) Levels of Ki-67 and PCNA in TPC-1 and IHH-4 transfected with sh-circ-ZNF609, tested utilizing Western blotting. All experimental results are recorded in the form of mean ± SD. *P < 0.05, **P < 0.01, ***P < 0.001 versus control or sh-NC.

To find out what the precise role of circ-ZNF609 was in TC, the detection of circ-ZNF609 expression in several TC cell lines was conducted. As shown in Figure 1(a), circ-ZNF609 expressed highly in TC cell lines CGTH-W3, TPC-1, IHH-4 CAL-62 and B-CPAP compared with that in normal thyroid cell-line Nthy-ori 3–1. TPC-1 and IHH-4 showed the higher expression of circ-ZNF609 among these TC cells. Thus, TPC-1 and IHH-4 cell lines were chosen as the subjects for follow-up experiments. To knockdown circ-ZNF609, the specific shRNA targeting circ-ZNF609 was then transfected into TPC-1 and IHH-4 cells, respectively. The interference efficiency of circ-ZNF609 was evaluated by the use of qRT-PCR (Figure 1(b)). As shown in Figure 1(c), the findings revealed that circ-ZNF609 silencing remarkably repressed the proliferation of TPC-1 and IHH-4 cells (vs Control). Also, the colony number of TPC-1 and IHH-4 cells after circ-ZNF609 knockdown was notably less than that in negative control cells (Figure 1(d-e)). Furthermore, the intensity of fluorescence of Ki-67 in circ-ZNF609 silenced cells was weaker than control and negative control (Figure 1(f)). Consistently, data from Western blot displayed that in both TPC-1 and IHH-4 cells, protein levels of Ki67 and PCNA were suppressed after transfection with sh-circ-ZNF609 (Figure 1(g)). These findings imply that circ-ZNF609 showed an inhibition in TC cell proliferation.

### Circ-ZNF609 knockdown restrains TC cell migration and invasion

**Figure 2.**
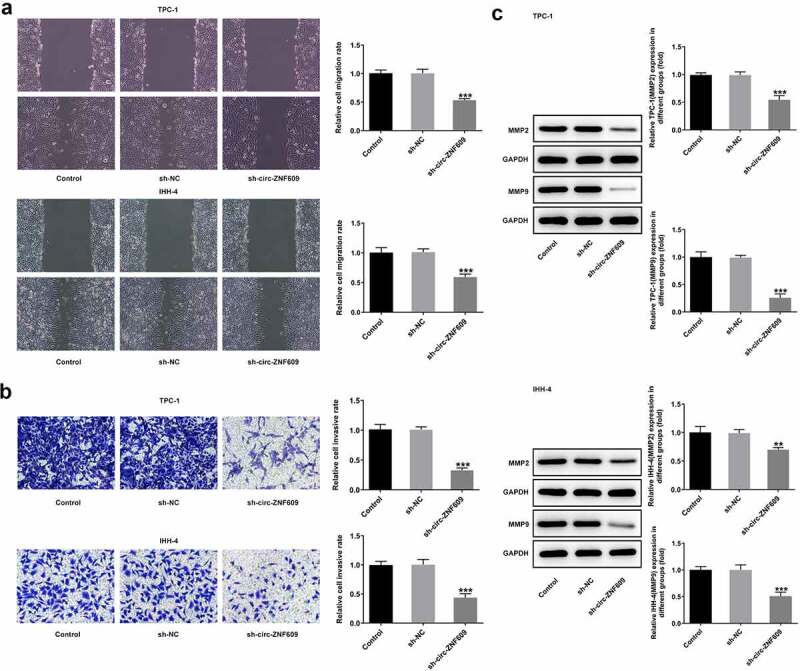
Circ-ZNF609 silencing suppressed the migration and invasion in TPC-1 and IHH-4 cells. (a) Cell migration detection employed wound healing following circZNF609 knockdown. Original magnification: 100 × . (b) Cell invasion was tested via transwell assay following circZNF609 knockdown. Original magnification: 100 × . (c) Levels of MMP2 and MMP9 following circZNF609 knockdown, examined with the aid of Western blotting. All experimental results are recorded in the form of mean ± SD. **P < 0.01, ***P < 0.001 versus sh-NC.

Cell migration capacity of TPC-1 was dramatically undermined by transfected with sh-circ-ZNF609 after 24-h incubation, as was that of IHH-4 cells (Figure 2(a)). In addition, the transwell assay showed that downregulation of circ-ZNF609 hindered TPC-1 and IHH-4 cell invasion by contrast with the negative controls (Figure 2(b)). Expectedly, protein expressions of MMP2 and MMP9 were obviously decreased after circ-ZNF609 was silenced in the two TC cell lines (Figure 2(c)), suggesting that the capacity of migration and invasion in TC cells was inhibited by circ-ZNF609 silencing.

### Circ-ZNF609 knockdown suppresses TC tumor growth in xenograft mouse model

**Figure 3.**
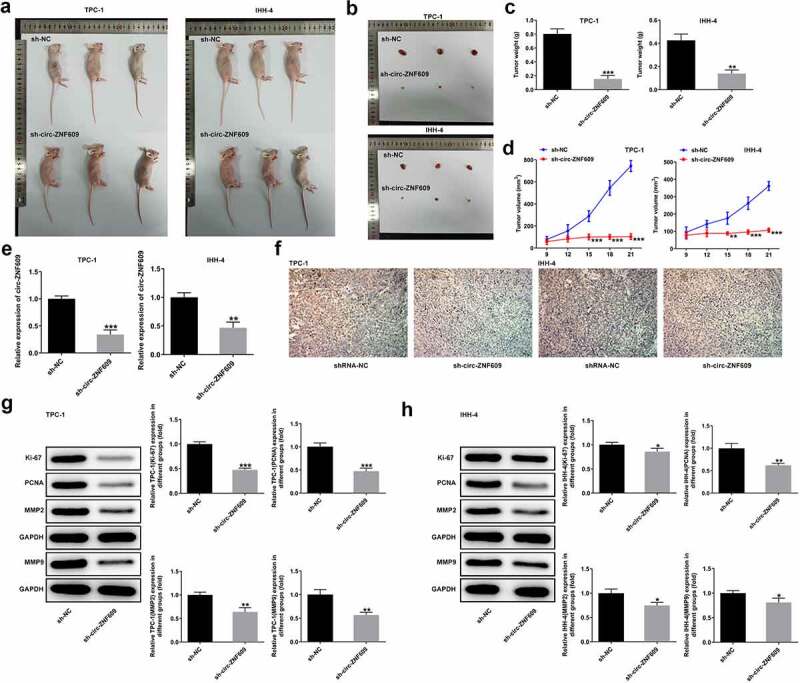
Circ-ZNF609 silencing inhibited tumor growth *in vivo*. (a-d) The tumor weight and volume of mice injected with sh-circ-ZNF609 were observed and measured. (e) Relative expression of circ-ZNF609 in tumor tissues, assayed through the way of qRT-PCR. (f) Immunohistochemistry experiments about Ki-67 in tumor tissue from the sh-circZNF609 mice. Original magnification: 200 × . (g-h) Levels of Ki-67 and PCNA in mice injected with TPC-1 and IHH-4 cells transfected with sh-circ-ZNF609, assessed with the adoption of Western blotting. All experimental results are recorded in the form of mean ± SD. *P < 0.05, **P < 0.01, ***P < 0.001 versus sh-NC.

The TPC-1 and IHH-4 cells after transfection with sh-circ-ZNF609 were prepared and injected into the flanks of nude mice subcutaneously, respectively. As shown in Figure 3(a-d), the sh-circ-ZNF609 group showed noticeably decreased weight and volume of tumors compared with the sh-NC group. Moreover, a low expression of circ-ZNF609 was examined in mice treated with cells transfected with sh-circ-ZNF609 (Figure 3(e)). The immunohistochemistry assay showed that Ki-67 expression was reduced in tumor tissues of nude mice treated with sh-circ-ZNF609 (Figure 3(f)). Moreover, decreased protein levels of Ki-67, PCNA, MMP2 and MMP9 were measured in the two TC cell lines after knockdown of circ-ANF609 (Figure 3(g, h)). These data indicate that circ-ZNF609 knockdown interfered TC growth *in vivo*.

### Circ-ZNF609 binds to miR-514a-5p

**Figure 4.**
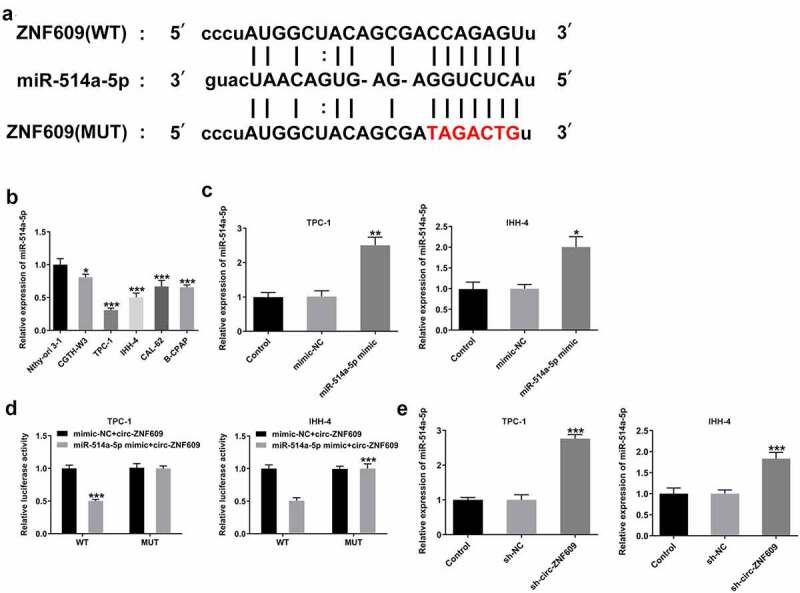
miR-514a-5p was sponged by circ-ZNF-609. (a) The binding site of circ-ZNF609 and miR-514a-5p. (b) Relative expression of miR-514a-5p in several TC cell lines and normal thyroid cell line Nthy-ori 3–1, examined with the employment of qRT-PCR. (c) Relative expression of miR-514a-5p in TPC-1 and IHH-4 cells transfected utilizing miR-514a-5p mimic. (d) Relative luciferase reporter activity was measured in cells co-transfected with miR-514a-5p mimics and wild type or mutant circ-ZNF-609. (e) Relative expression of miR-514a-5p in TC cells transfected with sh-circ-ZNF609, tested through the way of qRT-PCR. All experimental results are recorded in the form of mean ± SD. *P < 0.05, **P < 0.01, ***P < 0.001 versus control or sh-NC.

It has been demonstrated that circRNAs could act as ceRNAs to regulate downstream genes expression via sponging miRNAs [25]. To identify the mechanism underlying the modulation of circ-ZNF609 in TC cells, bioinformatics (https:// starbase.sysu.edu.cn/index.php) was utilized for searching the linked miRNAs that circ-ZNF609 sponges, and miR-514a-5p was found to have the binding site with circ-ZNF609 (Figure 4(a)). Then, we detected the expression of miR-514a-5p in TC cells and the results revealed that miR-514a-5p was significantly downregulated in several TC cell lines as compared to the normal thyroid cells (Figure 4(b)). MiR-514a-5p mimic was transfected into TPC-1 and IHH-4 cells for miR-514a-5p overexpression and the transfection efficiency was shown as Figure 4(c). As shown in Figure 4(d), dual-luciferase reporter assay of circ-ZNF609 and miR-514a-5p confirmed that miR-514a-5 mimic extremely inhibited the luciferase activity of circ-ZNF609-wt reporter in the two cell lines. Besides, miR-514a-5 expression was demonstrated to be elevated after inhibition of circ-ZNF609 (Figure 4(e)). The data suggest that circ-ZNF609 might sponge miR-514a-5p.

### Circ-ZNF609 silencing attenuates cell growth and metastasis by binding to miR-514a-5p

**Figure 5.**
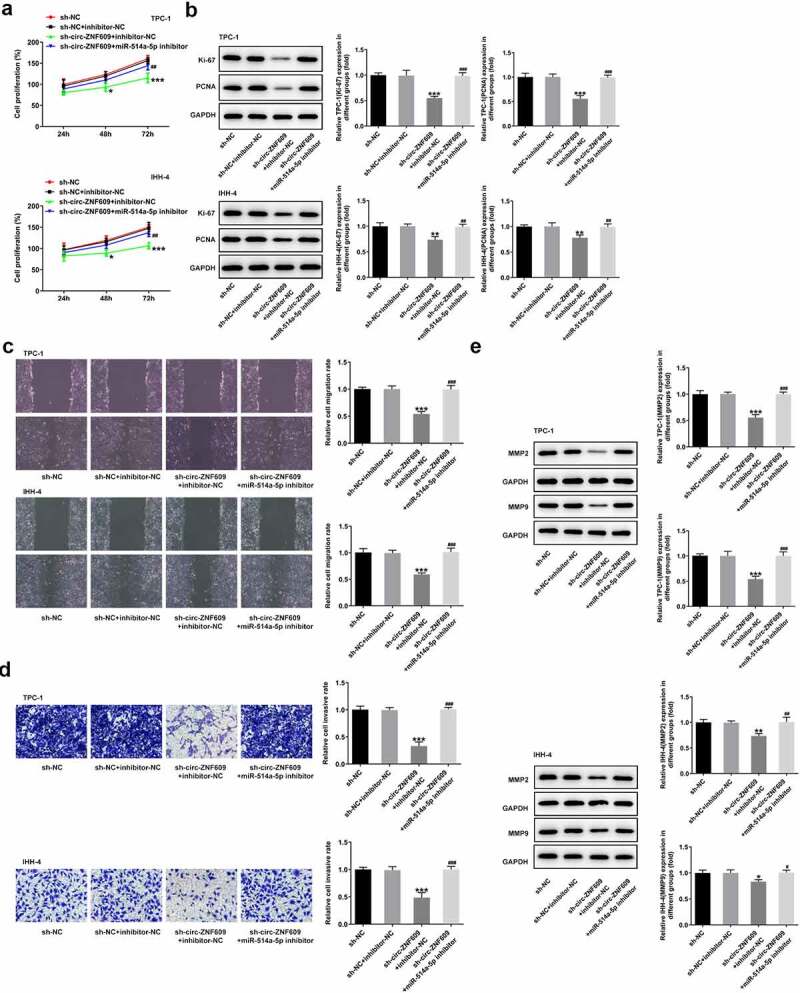
Circ-ZNF609 promotes cell growth and metastasis by down-regulating miR-514a-5p in TPC-1 and IHH-4 cells. (a) Cell proliferation assay employed the experiment of CCK-8. (b) Levels of Ki-67 and PCNA were assessed with the application of Western blot. (c) Cell migration evaluation was undertaken with the help of wound healing. Original magnification: 100 × . (d) Cell invasion experiment was carried out via the way of transwell. Original magnification: 100 × . (e) Levels of MMP2 and MMP9 was examined applying Western blotting. All experimental results are recorded in the form of mean ± SD. *P < 0.05, **P < 0.01, ***P < 0.001 versus sh-NC + inhibitor-NC. ^#^P < 0.05, ^##^P < 0.01, ^###^P < 0.001 versus sh-circ-ZNF609 + inhibitor-NC.

To test the role of combination with circ-ZNF609 and miR-514a-5p in TC development, we performed a rescue experiment to measure cell malignant biological behaviors. Figure 5(a) presents that cell proliferation in TPC-1 and IHH-4 cells is specifically suppressed as circ-ZNF609 was interfered (vs Control). However, miR-514a-5p inhibitor reversed the effects of circ-ZNF609 silencing on TC cell growth. Similarly, an obvious decrease in the protein levels of Ki-67 and PCNA was observed in sh-circ-ZNF609 group while inhibiting miR-514a-5p reduced the inhibitory effects of circ-ZNF609 silencing (Figure 5(b)). In addition, wound healing and transwell assay revealed that circ-ZNF609 inhibition remarkably restrained the migration and invasion of TPC-1 and IHH-4 cells, whereas miR-514a-5p inhibitor rehabilitated the weakening effect of circ-ZNF609 knockdown (Figure 5(c-d)). As expected, the contents of MMP2 and MMP9 were decreased in sh-circ-ZNF609 group, but the suppressive effects were reversed by silencing of miR-514a-5p (Figure 5(e)). The finding indicated that silencing of circ-ZNF609 abolished cell proliferation and metastasis by sponging miR-514a-5p.

### Downregulation of circ-ZNF609 hinderes TC tumor growth via binding to miR-514a-5p

**Figure 6.**
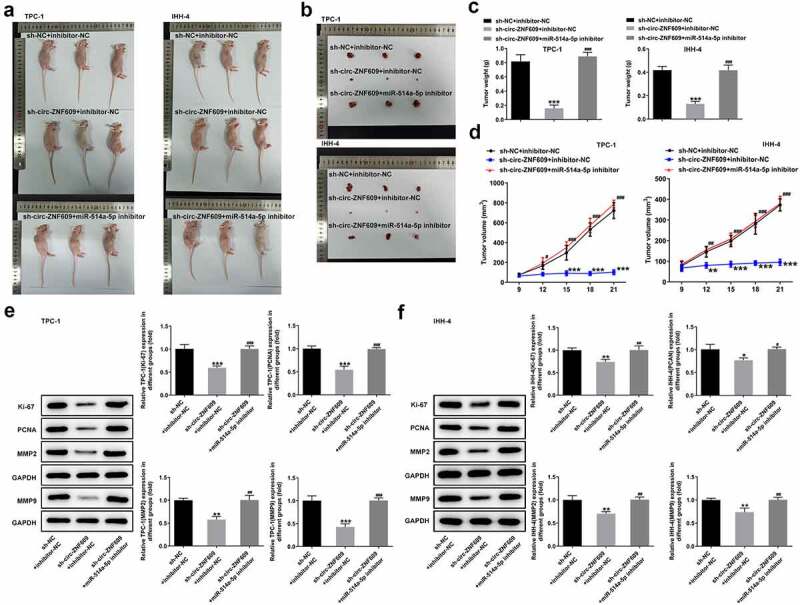
Circ-ZNF609 facilitated tumor growth by down-regulating miR-514a-5p *in vivo*. (a-d) The tumor weight and volume of mice treated with sh-circ-ZNF609 in the presence or absence of miR-514a-5p inhibitor were observed and measured. (e-f) Levels of Ki-67 and PCNA in TPC-1 and IHH-4 cells transfected with sh-circ-ZNF609 in the presence or absence of miR-514a-5p inhibitor, assayed employing Western blotting. All experimental results are recorded in the form of mean ± SD. *P < 0.05, **P < 0.01, ***P < 0.001 versus sh-NC + inhibitor-NC. ^#^P < 0.05, ^##^P < 0.01, ^###^P < 0.001 versus sh-circ-ZNF609 + inhibitor-NC.

Finally, we studied the functional role of interaction with circ-ZNF609 and miR-514a-5p in TC *in vivo*. As shown in Figure 6(a-d), the tumor weight and volume in mice treated with sh-circ-ZNF609 were greatly inhibited compared with the mice treated with the negative plasmids. Nevertheless, the inhibitory effects on tumor growth were abated by miR-514a-5p inhibitor. Additionally, miR-514a-5p silencing was revealed to promote the tumor weight and volume in sh-circ-ZNF609 treated mice (Figure 6(e, f)). Overall, the silencing of circ-ZNF609 markedly suppressed TC growth *in vivo* by binding to miR-514a-5p.

## Discussion

As a common endocrine malignancy worldwide with quickly increased incidence, TC has imposed a heavy medical burden on individuals and society [26]. Although numerous oncogene and tumor suppressor gene associated with TC have been revealed, the in-depth molecular mechanism is unknown [27–29]. In this study, we demonstrated that the knockdown of circ-ZNF609 not only repressed the malignant biological behaviors of TC *in vitro* but also suppressed the growth of tumors *in vivo*. Further mechanistic investigation revealed that miR-514a-5p was sponged by circ-ZNF609 and its silencing had inverse effects on TP cell proliferation and metastasis, and cancer growth in xenograft mice model.

Extensive research has established that circRNAs posed a vital role in the origination and development of human cancers [30] like oral breast cancer [31], bladder cancer [32], non-small cell lung cancer [33] and so on. Circ-ZNF609, a new-found circRNA homologous to ZNF609 mRNA, has exhibited tumor-promoting effects on the progressions of multiple types of cancers [34,35]. For instance, circ-ZNF609 facilitated carcinogenesis of gastric cancer cells through the inhibition of miRNA-145-5p expression [36]. Liao et al. reported that depletion of circ-ZNF609 repressed hepatocellular carcinoma (HCC) cell malignant biological behaviors while enhancing apoptosis, and circ-ZNF609 knockdown inhibited tumorigenesis of HCC mice [37]. A recent study showed that circ-ZNF609 promoted cervical cancer progression as an oncogene via the regulating E2F transcription factor 6 through competitively binding to microRNA-197-3p [38]. However, the mechanism of circ-ZNF609 in TC still needs more investigation. We then evaluated the functional role of circ-ZNF609 in TC. In our study, it was clearly observed that circ-ZNF609 was overexpressed in TC cells and circ-ZNF609 knockdown alleviated TC cell malignant biological behaviors. Ki-67 and PCNA are proliferating markers, while MMP2 and MMP9 are migration and invasion markers [39,40]. Our results indicated that circ-ZNF609 silencing significantly inhibited the levels of Ki-67, PCNA, MMP2 and MMP9 in TPC-1 and IHH-4 cell lines. *In vivo*, animal experiments displayed that depletion of circ-ZNF609 restrained the tumor volume and weight from mice, and reduced proliferation-related protein levels in tumor tissues, suggesting that circ-ZNF609 functioned as an oncogenic role in TP progression.

It is well documented that circRNAs act as ceRNAs to sponge miRNAs, so that they could exert a regulatory effect on the expressions of target genes downstream [41]. It has previously been reported that circ-ZNF609 could modulate cancer progression through sponging miRNAs [42]. Wang et al. revealed that circRNA ZNF609 promoted nasopharyngeal carcinoma angiogenesis by regulating the microRNA (miR)-145/Stathmin 1 (STMN1) axis [43]. Another study showed that knockdown of circZNF609 inhibited cell malignant biological behavior but facilitated apoptosis and repressed xenograft tumor growth in development of non-small cell lung cancer via regulating miR-623 and FOXM1 [44]. In this work, the binding sites of circ-ZNF609 and miR-514a-5p was predicted on the basis of data from bioinformatics analysis. Then, miR-514a-5p was expressed at a lower level in several TC cell lines. Meanwhile, we also revealed that circ-ZNF609 could bind to and negatively regulate miR-514a-5p in TC cells by luciferase reporter assay and qRT-PCR assay. A recent study demonstrated that LncRNA SNHG7 bound with miR-514a-5p and negatively modulated miR-514a-5p expression, and miR-514a-5p deficiency restored the progression of nasopharyngeal carcinoma inhibited by the knockdown of SNHG7 [45]. Rescue experiments indicated that inhibition of miR-514a-5p improved the ability of malignant biological behaviors of TC cells while reversing the effects on TC cells by circ-ZNF609, which was in line with the research published before. Additionally, mouse experiments with circ-ZNF609 knockdown with or without miR-514a-5p depletion showed that tumor growth was promoted after miR-514a-5p was knocked down. All of these data illustrated that circ-ZNF609 regulated TC development by binding to miR-514a-5p.

However, there are several limitations in this study. We mainly focus on the interaction between circ-ZNF609 and miR-514a-5p, but not explored the downstream target of miR-514a-5p. In addition, the signaling pathways triggered by circ-ZNF609 sponging miR-514a-5p in regulating TC cell development and metastasis need to be studied. We will further investigate downstream targets or pathways of circ-ZNF609/miR-514a-5p in TC in further study.

## Conclusion

To sum up, circ-ZNF609 functions as an oncogene in TC development via sponging miR-514a-5p, promoting cell proliferation and migration *in vitro* and accelerating tumor growth *in vivo*, which provides new insights into the understanding and implementing molecular-targeted treatment for TC.
